# 

**Published:** 1923-10

**Authors:** 


### RECENT APPOINTMENTS.

### Medical Officers, etc.

Bird, G. W. Harvey, M.R.C.S., L.R.C.P., M.O.H., Borough of Bridgwater.

Candon, Attractar Genevieve, M.B., B.Ch., Assistant School M.O., Sheffield Education Committee (Dublin).

Churchill, Stella, M.R.C.S., L.R.C.P., D.P.H., Assistant M.O., St. Pancras; Acting M.O., St. Pancras.

Clark, T. A. McDonald, M.D., D.P.H., M.O.H. and School Doctor, Walsall, £900.

Langton Peregrine Stephen Brackenbury, M.R.C.S., L.R.C.P., Deputy Medical Superintendent and Assistant M.O., York City Mental Hospital; Medical Superintendent, Royal Ediswood Institution for Mental Defectives, Earlswood.

Lloyd, Evan R., M.O.H., Uwchaled, Cerregydwidien and Llangwm.

Mackenzie, Ella, M.B., Assistant M.O.H., Blackburn.

O'Halloran, Anna, M.B., B.A., D.P.H., Assistant M.O.H., Bromley (Dublin).

Tarver, Mary L. (Queen Victoria's Jubilee Nursing Institute), Superintendent Huddersfield Maternity Branch.

Wardle, Isabella, C.M.B., Health Visitor, Durhan County Council.

Wenyon, C. M., C.M.G., C.B.E., M.B., B.Sc., Director of Research in the Tropics at the Wellcome Bureau of Scientific Research; Director in Chief of the Wellcome Bureau of Scientific Research.

### Public Health and Hospital Appointments.

Cantlow, Ella, House Sister, Hampstead General Hospital; Matron, St. Peter's Hospital, Henrietta Street, W.C.

Chittenden, A. E. Miss, Health Visitor, Essex County Council.

Collins, Sylvia, Health Visitor, Bristol; Health Visitor, Taunton.

Dakin, Stella; Health Visitor, Stoke-on-Trent.

Dempster, Margaret W., Health Visitor, Manchester.

James, Martha A., District Nurse, Pontlothyn; Health Visitor, Monmouthshire County Council.

Platt, Mabel, Health Visitor, Willesden.

Richards, F. J., Sanitary Inspector, Gellygaer.

Rogers, C. D., Sister Housekeeper, Swansea General and Eye Hospital; Assistant Matron, Royal Sussex County Hospital.

Snow, Daisy S., Health Visitor, Monmouthshire County Council.

Taylor, Annis, Health Visitor for Leicestershire; Health Visitor Stoke-on-Trent.

Traynor, Edith B., Health Visitor, Manchester.

White, R. H., Borough Surveyor and Sanitary Inspector, Richmond (Yorkshire).

Many shampoo powders possess a strong scent which may in time deteriorate into a rather unpleasant odour. The manufacturers of the "Amami" shampoo have been careful to avoid this danger, and those who use their powders will find them delicately scented with a perfume which is never obtrusive. In addition the "Amami" shampoo powder may be thoroughly recommended for its cleansing and refreshing qualities.

